# Advancing the Care Experience for patients receiving Palliative care as they Transition from hospital to Home (ACEPATH): Codesigning an intervention to improve patient and family caregiver experiences

**DOI:** 10.1111/hex.14002

**Published:** 2024-03-28

**Authors:** Madeline McCoy, Taylor Shorting, Vinay Kumar Mysore, Edward Fitzgibbon, Jill Rice, Meghan Savigny, Marianne Weiss, Daniel Vincent, Meaghen Hagarty, Krystal Kehoe MacLeod, Natalie C. Ernecoff, Rex Pattison, Mona Kornberg, Adrianna Bruni, Shirley H. Bush, Kerry Kuluski, Valerie Fiset, Cecilia Li, Henrique A. Parsons, Geneviève Lalumière, Tara Connolly, Colleen Webber, Sarina R. Isenberg

**Affiliations:** ^1^ Bruyère Research Institute Ottawa Ontario Canada; ^2^ Parsons School of Design, The New School New York New York USA; ^3^ OpenBox Brooklyn New York USA; ^4^ The Ottawa Hospital Ottawa Ontario Canada; ^5^ Bruyère Continuing Care Ottawa Ontario Canada; ^6^ Department of Medicine, Division of Palliative Care University of Ottawa Ottawa Ontario Canada; ^7^ Ottawa Hospital Research Institute Ottawa Ontario Canada; ^8^ Consultant; ^9^ Marquette University Milwaukee Wisconsin USA; ^10^ Department of Family Medicine University of Ottawa Ottawa Ontario Canada; ^11^ RAND Corporation Pittsburgh Pennsylvania USA; ^12^ Caregiver Partner; ^13^ Institute for Better Health, Trillium Health Partners Mississauga Ontario Canada; ^14^ Institute of Health Policy, Management and Evaluation University of Toronto Toronto Ontario Canada; ^15^ Champlain Hospice Palliative Care Program Ottawa Ontario Canada; ^16^ School of Nursing, University of Ottawa Ottawa Ontario Canada; ^17^ Regional Palliative Consultation Team (RPCT) Ottawa Ontario Canada; ^18^ Accessibility Institute Carleton University Ottawa Ontario Canada; ^19^ School of Epidemiology and Public Health University of Ottawa Ottawa Ontario Canada

**Keywords:** care transitions, codesign, health services research, palliative care, patient and caregiver engagement

## Abstract

**Background:**

Returning home from the hospital for palliative‐focused care is a common transition, but the process can be emotionally distressing and logistically challenging for patients and caregivers. While interventions exist to aid in the transition, none have been developed in partnership with patients and caregivers.

**Objective:**

To undergo the initial stages of codesign to create an intervention (Advancing the Care Experience for patients receiving Palliative care as they Transition from hospital to Home [ACEPATH]) to improve the experience of hospital‐to‐home transitions for adult patients receiving palliative care and their caregiver(s).

**Methods:**

The codesign process consisted of (1) the development of codesign workshop (CDW) materials to communicate key findings from prior research to CDW participants; (2) CDWs with patients, caregivers and healthcare providers (HCPs); and (3) low‐fidelity prototype testing to review CDW outputs and develop low‐fidelity prototypes of interventions. HCPs provided feedback on the viability of low‐fidelity prototypes.

**Results:**

Three patients, seven caregivers and five HCPs participated in eight CDWs from July 2022 to March 2023. CDWs resulted in four intervention prototypes: a checklist, quick reference sheets, a patient/caregiver workbook and a transition navigator role. Outputs from CDWs included descriptions of interventions and measures of success. In April 2023, the four prototypes were presented in four low‐fidelity prototype sessions to 20 HCPs. Participants in the low‐fidelity prototype sessions provided feedback on what the interventions could look like, what problems the interventions were trying to solve and concerns about the interventions.

**Conclusion:**

Insights gained from this codesign work will inform high‐fidelity prototype testing and the eventual implementation and evaluation of an ACEPATH intervention that aims to improve hospital‐to‐home transitions for patients receiving a palliative approach to care.

**Patient or Public Contribution:**

Patients and caregivers with lived experience attended CDWs aimed at designing an intervention to improve the transition from hospital to home. Their direct involvement aligns the intervention with patients' and caregivers' needs when transitioning from hospital to home. Furthermore, four patient/caregiver advisors were engaged throughout the project (from grant writing through to manuscript writing) to ensure all stages were patient‐ and caregiver‐centred.

## 1 INTRODUCTION

We were never told how long [my husband] would have to remain in hospital. He was discharged with little information. He had walked into the hospital managing his personal care. He left completely dependent. I was unprepared. I felt I was going home blind! I had no direction as to what to expect before and after we came home. I am struck by the poor coordination and the system that is not patient‐centred, nor supportive of caregivers.

This quote from a caregiver advisor whose husband transitioned from hospital to home while receiving palliative care (PC) provides insights into the challenges patients and their caregivers (caregivers are defined as people who provide informal support to a patient and do not need to be directly related to the patient [i.e., family member, friend, neighbour]) experience during this transition.

### 1.1 Hospital‐to‐home transitions in PC

PC is an approach to care, often delivered by an interprofessional team, aimed at reducing symptom burden and addressing the physical, psychological, spiritual and emotional needs of patients with life‐limiting illnesses.^1^ In Canada, PC is provided across settings: hospital‐based services, including PC units, specialist consultation teams and outpatient clinics; and community services, including PC provided in patients' homes (private or residential care facilities), and residential hospices.^2^

As people with life‐limiting illnesses approach end of life, many consider the possibility of receiving PC at home.^3^ American and European studies have shown that approximately 50%–60% of patients experience a transition across care settings during the last 4 weeks of life.^4^, ^5^ This current study considered transitions from hospital to home, retirement home or group home. Hospital‐to‐home transitions in PC can be distressing and logistically challenging,^6^ with significant negative effects on the well‐being of patients and caregivers.^7^, ^8^, ^9^, ^10^, ^11^, ^12^ Few studies have focused on hospital‐to‐home transitions for patients receiving palliative‐focused care.

### 1.2 Shortcomings of existing interventions

Previous work examining hospital‐to‐home transitions for patients receiving PC included several studies evaluating transition interventions and one systematic review.^10^, ^11^, ^13^, ^14^, ^15^, ^16^ This review found that most interventions involved a care coordinator, used hospital readmissions as the primary outcome and demonstrated reduced readmission rates.^11^ However, the review included studies at high risk of bias, most commonly due to insufficient length of follow‐up or missing data.^11^ Other literature focused on interventions to reduce hospital readmission rates for patients receiving PC at home by deploying nurse navigators or care coordinators, revealing mixed results.^17^, ^18^, ^19^, ^20^ While previous interventions showed some benefits, these studies had not vetted their outcomes for importance to patients and caregivers.^17^, ^18^, ^19^, ^20^ Without patient and caregiver involvement, interventions may not capture the components they most value to improve outcomes.

### 1.3 Codesign as a patient‐ and caregiver‐centred approach

Research involving codesign processes has engaged diverse stakeholders in intervention development. Codesign can involve researchers, designers, clinicians, patients and caregivers, with patients and caregivers central in the process.^21^ By providing the opportunity for patients and caregivers to share lived experiences throughout concept development, codesigned interventions are more likely to respond successfully to their needs.^21^ Codesign has effectively been used for inpatient care studies with favourable results^22^ and has been applied to various improvement initiatives, including transitions across care settings, home care and initiatives for older adults and patients receiving PC.^23^, ^24^, ^25^, ^26^, ^27^ When patients and caregivers have been involved in participatory research, they reported a sense of being heard^24^ and favourable change towards collaboration and ownership.^28^ Furthermore, the engagement of patients and caregivers has created opportunities to challenge long‐standing concerns in medicine where the voices of healthcare providers (HCPs) take precedence over those with lived experience.^29^ None of the hospital‐to‐home interventions were developed using a codesign approach with patients and caregivers. We have applied the codesign approach to the PC space, which has not been done in the Canadian context.

### 1.4 Objective

By leveraging the codesign approach, the objective was to undergo the initial stages of codesign to create an intervention (Advancing the Care Experience for patients receiving Palliative care as they Transition from hospital to Home [ACEPATH]), informed by patients, caregivers and HCPs, that was tailored to the needs of the Canadian hospital‐to‐home transition process, while ensuring adaptability to local contexts.

### 1.5 Setting

The setting was The Ottawa Hospital, a large tertiary academic hospital in Ottawa, Ontario, Canada. The study site had an interprofessional PC consultation team, including physicians, nurses and social workers. In 2022, the team found that 28% of the 3541 patients seen in consultation were discharged home. Among those discharged home, the mean time to death was 34 days. When a patient transitions from hospital to home, the PC team assists the attending team to coordinate care and facilitate the discharge. Discharges from this hospital were complex, and health utilization outcomes (e.g., readmissions, emergency department visits) suggested transitions were suboptimal.

## 2 METHODS

**Figure 1:**
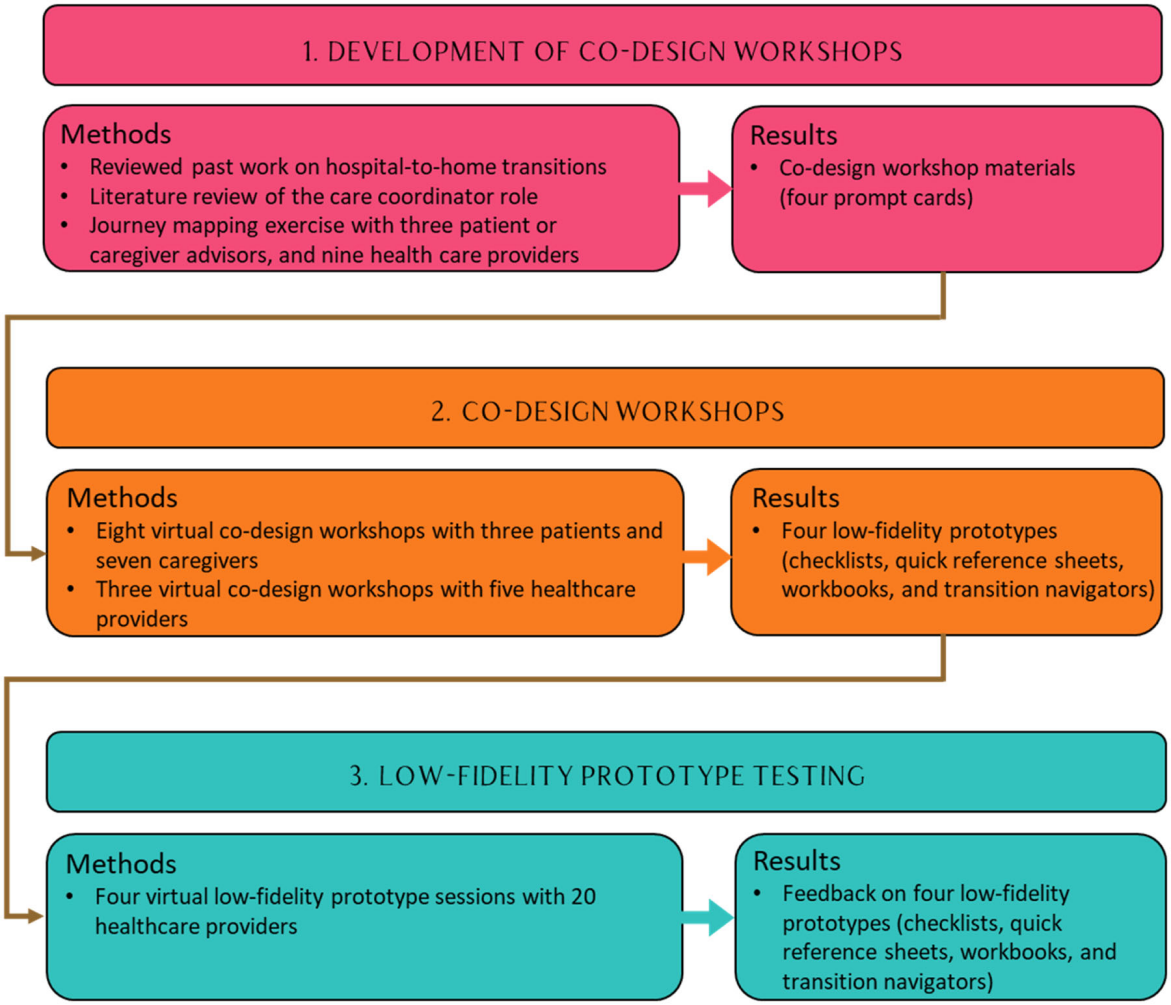
Codesign process stages: development of codesign workshops, codesign workshops and low‐fidelity prototype testing.

The codesign process consisted of (1) development of codesign workshops, (2) codesign workshops and (3) low‐fidelity prototype testing^10^, ^13^, ^14^, ^30^, ^31^, ^32^, ^33^ (see Figure 1 for an overview of the study). The study was reviewed by a research ethics board and granted exemption because it met the criteria of a quality improvement project. However, we incorporated ethical considerations into the study design, such as clarifying that participation was voluntary, outlining steps to be taken if patients and caregivers became uncomfortable or distressed during the workshops and ensuring accessibility throughout the study. The core research team consisted of the health services researcher, PC physicians and nurses, the design researcher, the communications designer and research support staff.

**Figure 2:**
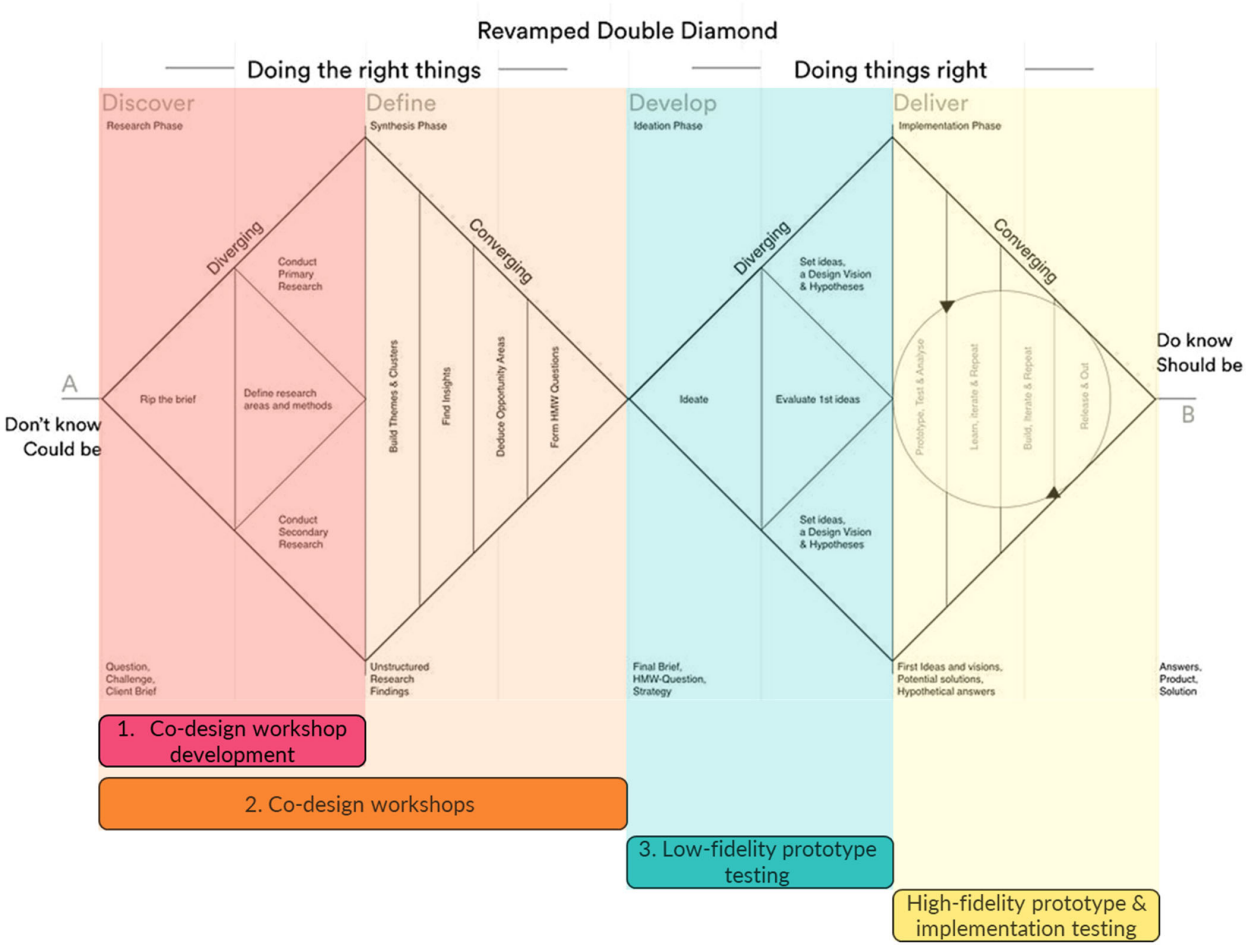
The Double Diamond Framework with the steps in the codesign process mapped on.^31^, ^32^, ^33^

The codesign process followed the Double Diamond Framework, developed by the Design Council UK in 2005^32^, ^33^ and revamped in 2018 as a graphical way to describe the steps in a design process.^31^ Using this framework, there were four stages to the design process.^31^, ^32^, ^33^ See Figure 2 for a visual depiction of the design process and how our project stages map onto the framework. The first diverging phase (Discover—Research phase) collected the necessary information to understand the problem via the codesign development phase and the codesign workshops and determined the direction and methods for codesign workshops.^31^, ^32^, ^33^ The second converging phase (Define—Synthesis phase) used affinity mapping^34^ to synthesize findings from previous research and codesign workshops.^31^, ^32^, ^33^ The third diverging phase (Develop—Ideation phase) generated ideas for potential interventions and explored how they could be tangible.^31^ The research team created sketches and storyboards of prototypes. The final fourth phase will involve another converging phase (Deliver—Implementation phase) and result in the delivery of the final ACEPATH intervention for implementation (more detail in Section 4.3).

### 2.1 Stage 1: Development of codesign workshops

Journey mapping^35^, ^36^, ^37^ was used to visualize the processes individuals experience as they transition from hospital to home. The aim was to clarify and communicate key findings from prior research^10^, ^13^, ^14^, ^15^, ^30^ and reflect on the emotions, needs and experiences of patients and caregivers as they navigate the hospital‐to‐home transition. The journey mapping exercise helped develop key communication and research assets including materials and prompts for the codesign workshops.

#### 2.1.1 Recruitment

An email was sent to project coinvestigators and collaborators including HCPs and patient/caregiver advisors.

#### 2.1.2 Data collection

Journey maps could entail a list of steps, an instruction manual, a story or a voice recording, memo or dictation detailing the journey from hospital to home. Participants were asked to describe, in their own words, the patient journey from hospital to home, including the steps required to get home, when these steps happen, and HCPs or other stakeholders involved in the transition. Participants also described what could happen if a patient returns to the hospital after discharge home.

#### 2.1.3 Analysis of journey maps

The team used affinity mapping^34^ to cluster journey maps into key moments during a patient's hospital‐to‐home transition that a codesigned intervention could address. The codesign workshop prompts elicited conversation on the challenges encountered at key moments in the transition journey and relevant solutions.

### 2.2 Stage 2: Codesign workshops

In the codesign workshops, the research team spoke with patients, caregivers and HCPs to understand challenges, identify metrics for successful transitions (covered in a subsequent manuscript), and identify tangible actions that would improve the patient and caregiver transition experience. Based on findings from the codesign development phase, the team determined three objectives for the workshops: identification of the greatest challenges in the hospital‐to‐home transition, key patient/caregiver metrics for success in hospital‐to‐home transitions, and tangible actions that would improve the patient and caregiver transition experience.

#### 2.2.1 Recruitment

The PC team or other HCPs from the patient's circle of care approached patients (or their caregiver) who had received an inpatient PC consultation, had comfort‐focused goals of care, and wanted to return home for PC. The HCP forwarded interested patients' and/or caregivers' contact information to the research coordinator (RC), who then provided patients and/or caregivers more information about the study.

The RC emailed site attending PC physicians weekly regarding recruiting potential participants. Additionally, the research team presented virtually to local and regional stakeholders regarding recruitment strategies, inclusion criteria and the process to contact the RC.

Potential patient and caregiver participants were screened by the RC to ensure they met the inclusion criteria:

1. Patients have experienced a hospital‐to‐home transition while receiving PC at the study site or have expressed a preference for receiving comfort‐focused care at home postdischarge.
2. Patients have a Palliative Performance Scale between 30% and 50% (30% representing a bedbound patient).^38^
3. Patients and caregivers can communicate in English or French and are above the age of 18.

We aimed to include a diverse representation of patients and caregivers in the workshops. However, due to low recruitment numbers, all eligible potential participants were accepted. To increase diversity, the research team contacted 16 organizations in the area serving equity‐deserving groups, for example, those without English as a first language, with lower income and so forth. Three organizations shared the recruitment letter through their networks and one organization provided contact information for a potential participant.

Additionally, separate workshops were conducted with HCPs to share their experiences supporting diverse patients and caregivers in hospitals and/or the community (e.g., rural, low socioeconomic status and/or immigrant communities) to supplement the limited diversity of patient and caregiver participants.

Potential participants were screened by the RC to ensure they met the inclusion criteria:

1. HCPs are involved in the hospital‐to‐home transition for patients receiving PC at the study site and have identified that they support diverse patients and caregivers.
2. Participants can communicate in English or French and are above the age of 18.

#### 2.2.2 Data collection

Workshops were scheduled with patient and/or caregiver participants following discharge home. The RC obtained participants' home addresses to which they delivered four physical prompt cards, ensuring accessibility. Participants could write on the cards, make notes for the session and/or have materials available to prompt conversation during the workshop.

All workshops were 60‐min and virtual (using Zoom). Patients and caregivers could participate independently or in a patient/caregiver dyad. Workshops were audio‐ and video‐recorded and transcribed, and an RC took notes during workshops.

Codesign workshop participants were given a demographics questionnaire via Microsoft Forms which collected age, preferred language of communication, location (urban or rural), gender, status as Indigenous or a visible minority and identification as a patient or caregiver.

#### 2.2.3 Codesign workshop materials

Prompt cards were designed to facilitate conversations on each of the four key questions:

1. Is going home worth it? What makes it worthwhile?
2. What is essential to make care at home work?
3. Imagine someone who coordinates home transitions. What job responsibilities would be most helpful?
4. Think back to waiting in the hospital before discharge home. What is useful to know while in the hospital?

**Figure 3:**
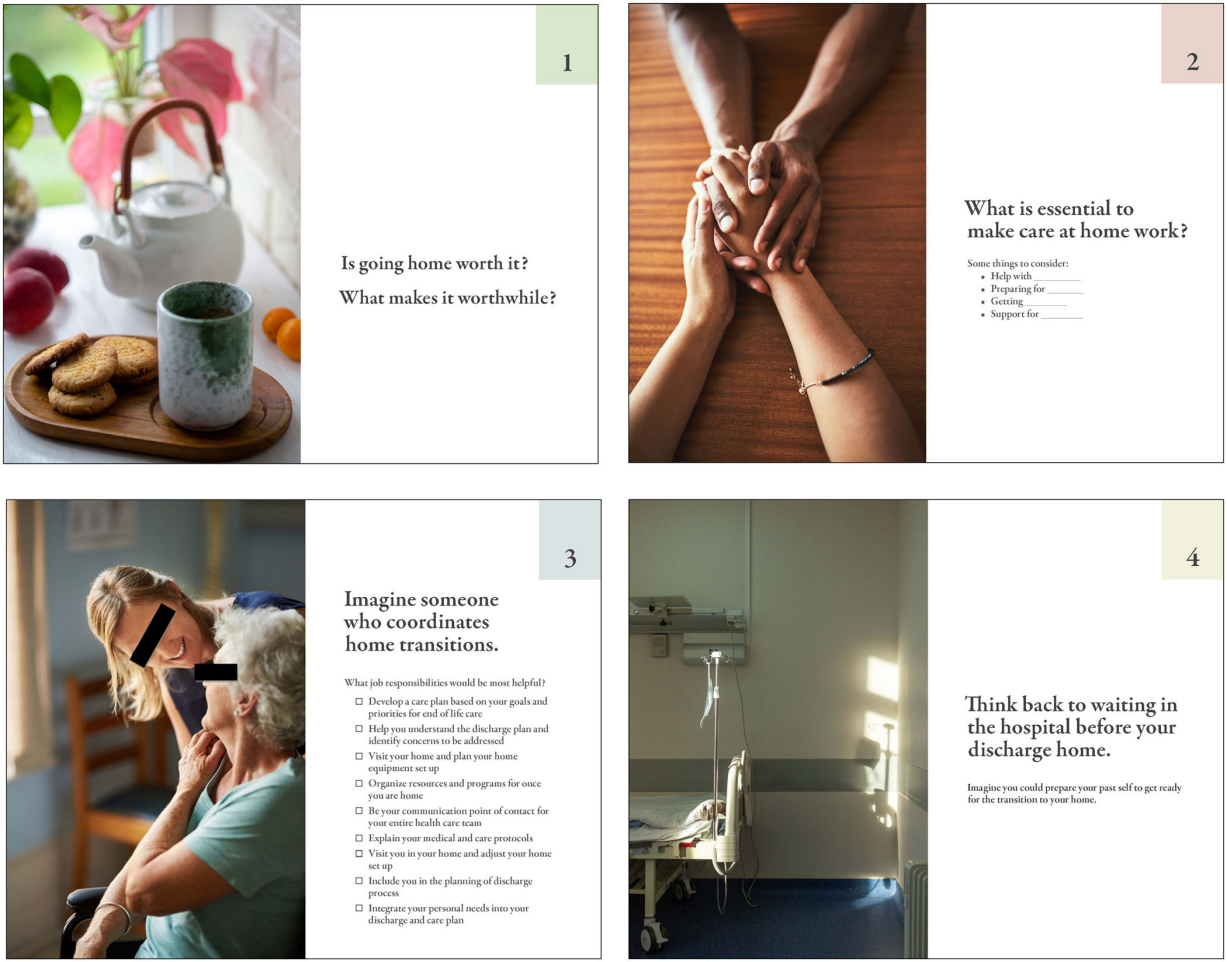
Initial prompt cards used in codesign workshops.

**Figure 4:**
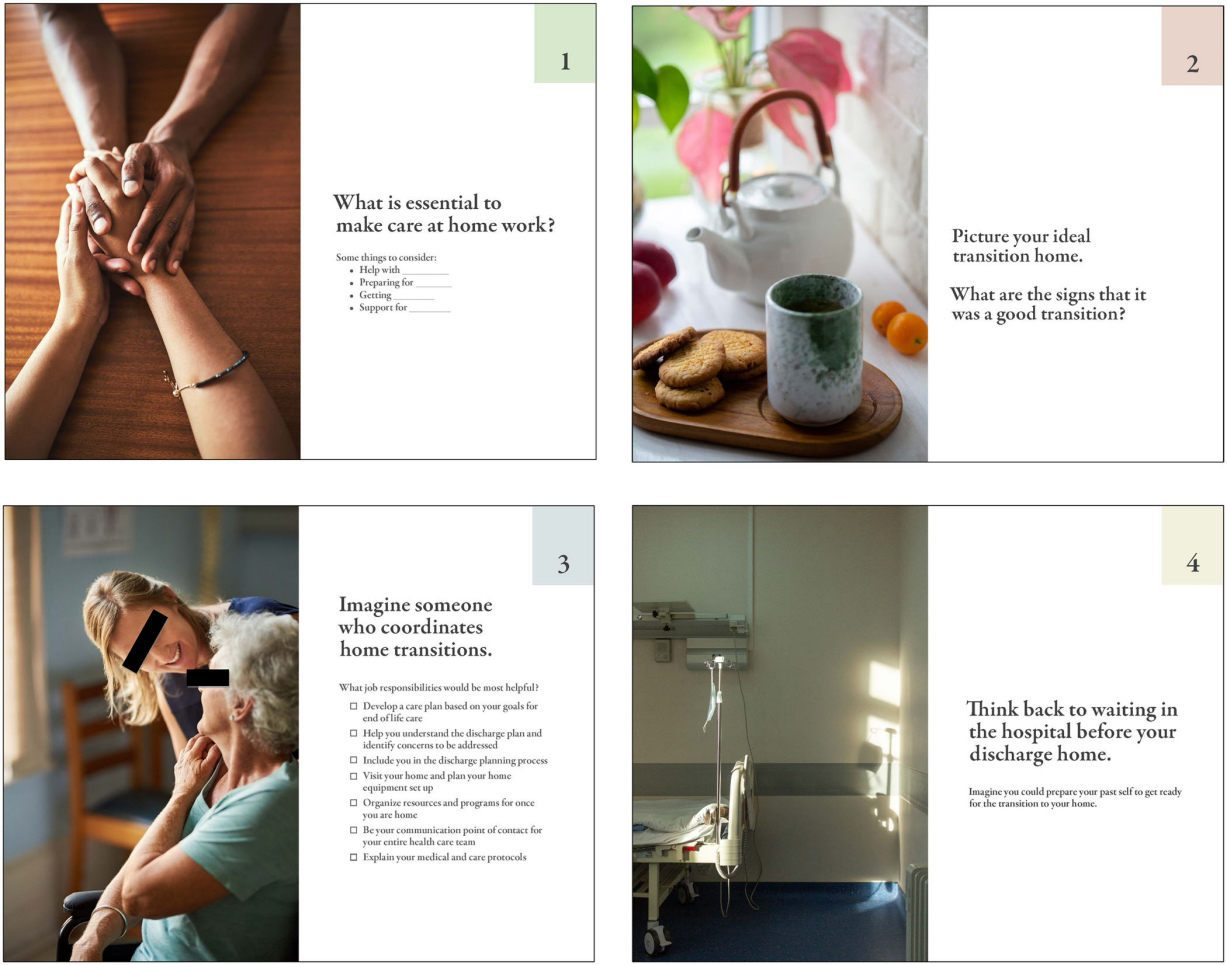
Revised prompt cards used in codesign workshops.

The cards were designed by a communications designer with accessibility considerations in mind. The cards were large and high‐contrast for those with limited vision and printed on cardstock for accessible handling.^39^, ^40^ The cards were numbered to facilitate use during the workshop activities and emotive images were used to prompt discussion. See Figure 3 for the initial prompt cards and Figure 4 for the revised prompt cards, which were adapted using an iterative process as workshops were conducted.

#### 2.2.4 Analysis of codesign workshops

Workshop transcripts and notes were uploaded into Miro,^41^ a program that facilitates collaboration between team members and visualization of themes using virtual sticky notes and text boxes.^34^, ^42^ The team reviewed the transcripts and notes from each workshop together and organized the quotes into themes.^42^ Themes and ideas were clustered, forming areas for action.^31^, ^42^

The research team created sketches and storyboards conceptualizing potential interventions, when the intervention might be used in the transition process, who would be involved in using or implementing the intervention, and how the intervention might provide a solution to the problems that were uncovered in the codesign workshops (see Supporting Information S1: Appendix A for examples of initial sketches). The research team met to discuss ideas, taking the design principles, patient and caregiver perspectives and system and process considerations into account. The design researcher and communications designer refined these ideas by combining elements into low‐fidelity prototypes.

### 2.3 Stage 3: Low‐fidelity prototype testing

The low‐fidelity prototypes were developed by the research team to display early ideation and testing of ideas (e.g., concept sketches, paper prototypes, storyboards).^43^ HCPs were included in low‐fidelity prototype testing to explore the applications of the interventions developed from the codesign workshops. These prototypes precede high‐fidelity prototypes, which are refined, and represent the look, feel and functionality of the final product (e.g., immersive roleplays, booklets).^43^

#### 2.3.1 Recruitment

The RC sent an email to invite coinvestigators, home care staff and HCP codesign workshop participants to a low‐fidelity prototype session. Coinvestigators forwarded the email to colleagues who coordinated discharge or cared for patients during the hospital‐to‐home transition.

#### 2.3.2 Data collection

The workshops were conducted virtually (Zoom) and were audio‐ and video‐recorded to produce a transcript of the sessions. Participants were invited to workshops separate from their direct supervisors to encourage participants to speak freely.

Workshop participants were shown a PowerPoint presentation that gave an overview of four low‐fidelity prototypes (see Figure 5) and the process used to synthesize prototypes from the codesign workshops. The design researcher led the workshops, encouraging participants to provide feedback on how these prototypes might fit into current scopes of practice, what interventions or parts thereof would be realistic to implement, and how the intervention could work in practice.

Figure 5Low‐fidelity prototypes presented at low‐fidelity prototype sessions consisting of checklist, quick reference sheets, patient/caregiver workbook and transition navigator.

**Figure:**
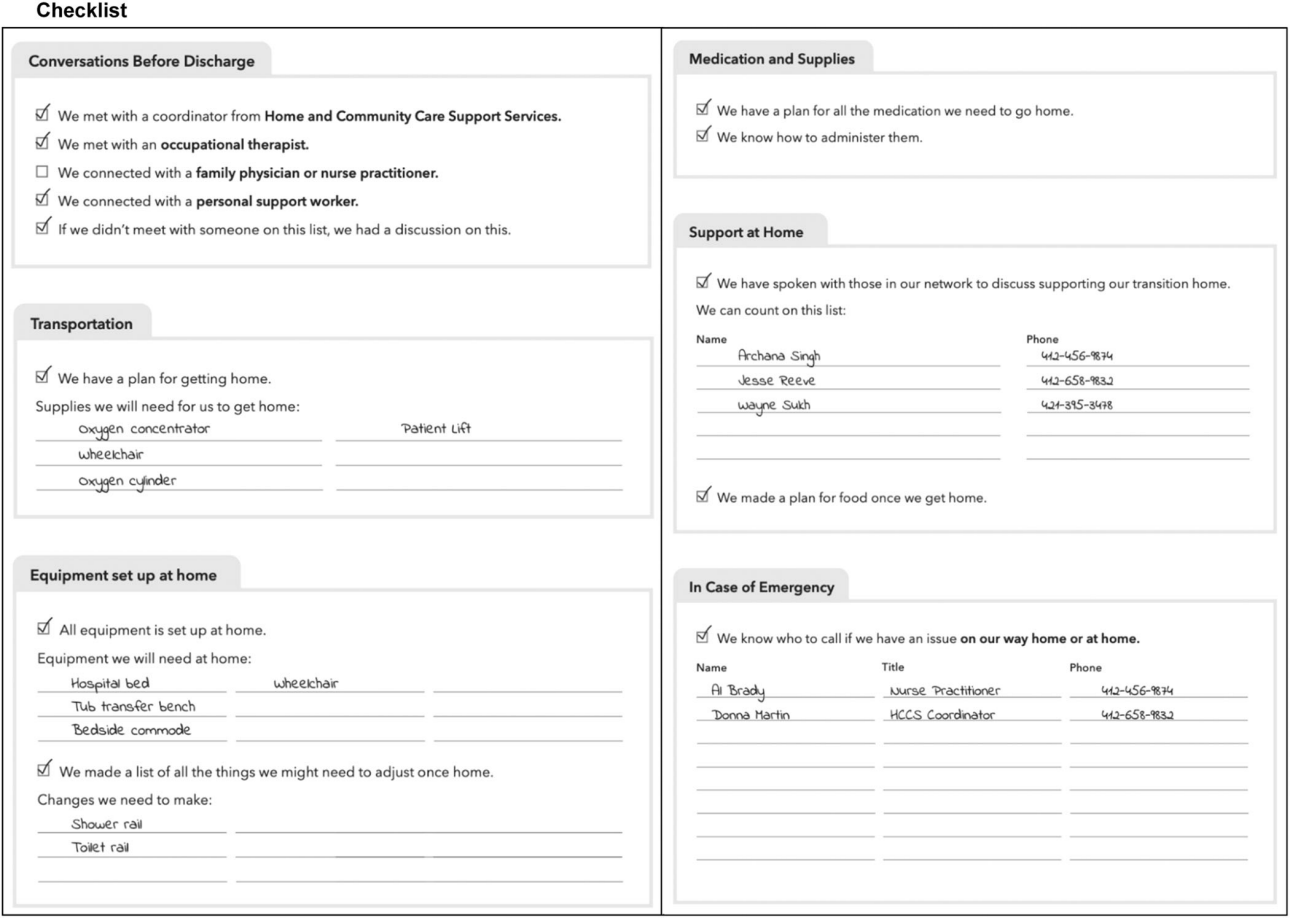

**Figure:**
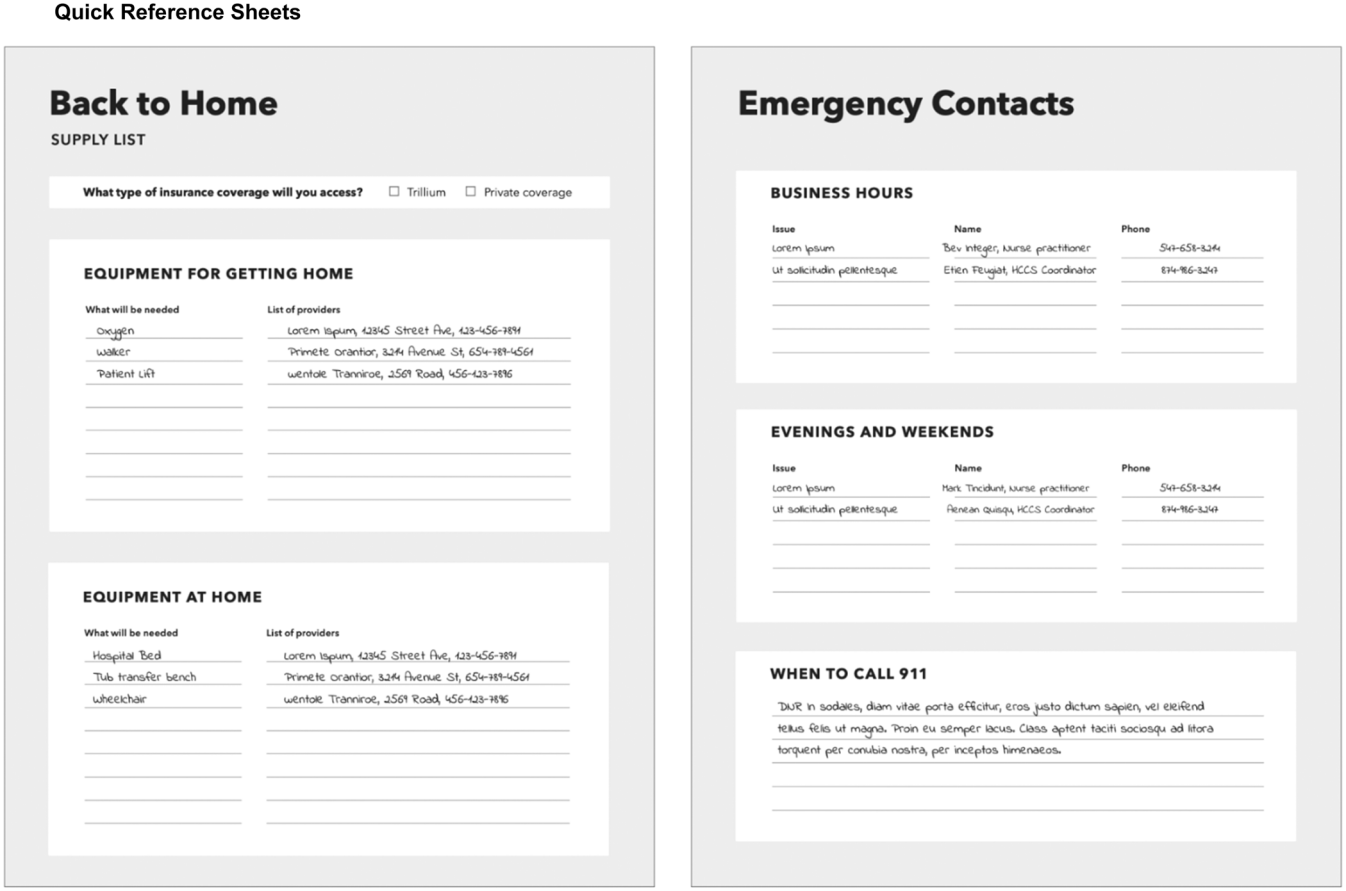

**Figure:**
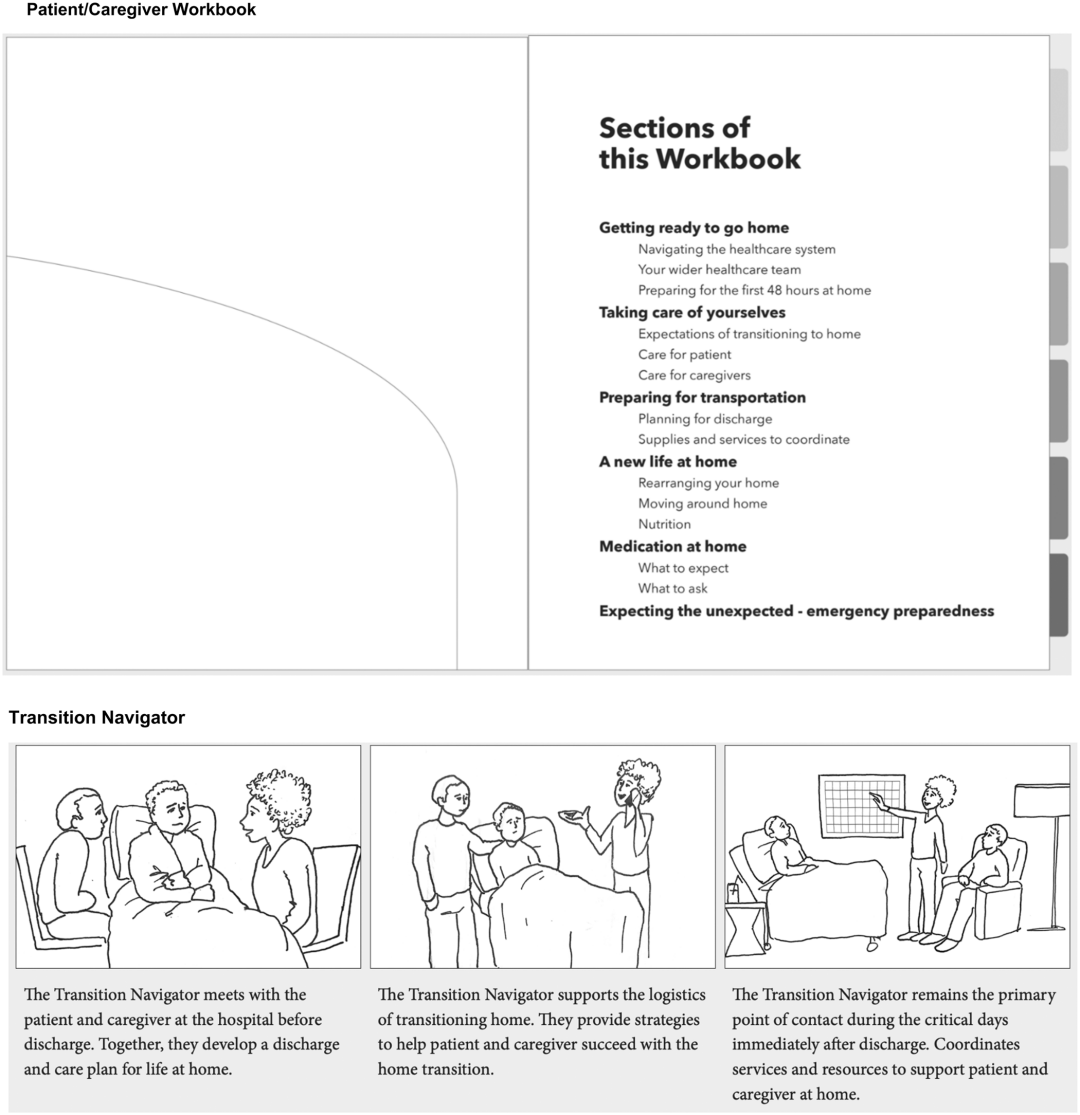

#### 2.3.3 Analysis of low‐fidelity prototype feedback

The research team consolidated feedback on each prototype. Affinity mapping^34^ was used to cluster notes into the following themes: what does the intervention look like?; what problem(s) is/are the intervention trying to solve?; and what are the concerns about the intervention?

## 3 RESULTS

### 3.1 Stage 1: Development of codesign workshops

Twelve participants completed the journey mapping exercise: five PC physicians (hospital or community), two nurse specialists or advanced practice nurses, two home care managers/directors and three patient/caregiver advisors.

After reviewing the journey maps, the team highlighted three key moments during patients' hospital‐to‐home transition: (1) Waiting in the hospital for discharge; (2) the first 48 hours after transitioning home; (3) longer‐term stabilization of care at home postdischarge.

Participants highlighted the administrative load experienced by caregivers while patients were waiting in the hospital for discharge. Conversations occurred between the PC team, social workers, home care and additional community teams, but communication between team members and taking responsibility for different elements of the discharge were noted to be challenging. Caregivers reported experiencing an additional administrative load as they were often responsible for coordinating many of the physical supports (e.g., setting up equipment, obtaining medical supplies) before discharge home.

When considering the first 48 hours after discharge home, the journey maps highlighted a dissonance between caregiver expectations and the actual experience of returning home. This included expectations around timelines for discharge, types of services available, frequency of contact or visits by HCPs and hours of home care support. For example, after returning home, caregivers subsequently realized that fewer personal support worker (PSW) hours would be provided than anticipated predischarge. Additionally, caregivers desired additional support by telephone in the period between discharge home and being contacted by home care coordinators or visited by a nurse or PSW. Notably, hours of care provided are reassessed frequently to adjust to changing needs but often remain insufficient for patients and caregivers.

Finally, following stabilization in care postdischarge, caregivers experienced additional financial expenses when government‐funded home care did not adequately meet patient needs. Thus, the incongruence between the expectation and the experience of going home was further highlighted, particularly when patients outlived their expected prognosis.

### 3.2 Stage 2: Codesign workshops

Three patients and seven caregivers participated in eight workshops, and five HCPs participated in three workshops.

Most patient and caregiver participants were 65+ years old (*n* = 7; 70%), lived in an urban area (*n* = 8; 80%), identified as women (*n* = 8; 80%) and were not Indigenous (*n* = 9; 90%) or a visible minority (*n* = 9; 90%). All patients and caregivers preferred to speak English, although one participant expressed that they were bilingual (English and French).

HCPs worked in the community and consisted of two physicians, two nurses and one nurse practitioner. Most of the HCPs also worked in an urban area (*n* = 4; 80%) and all preferred to speak English, although two were bilingual (English and French). The HCPs supported patients who lived in rural areas, Indigenous populations, Francophone populations, visible minorities, immigrant populations, refugee populations and people with lower income. Table 1 provides a summary of the participants in the codesign workshops.

**Table 1:** Participant table—codesign workshops and low‐fidelity prototype workshops.

Codesign workshops			
Patients (*n* = 3)		Caregivers (*n* = 7)	
	Number (%)		Number (%)
Age		Age	
Under 65	0 (0%)	Under 65	3 (43%)
Over 65	3 (100%)	Over 65	4 (57%)
Preferred language of communication		Preferred language of communication	
English	3 (100%)	English	7 (100%)
French	0 (0%)	French	0 (0%)
Location		Location	
Urban (more than 1000 people)	2 (67%)	Urban (more than 1000 people)	6 (86%)
Rural (less than 1000 people)	1 (33%)	Rural (less than 1000 people)	1 (14%)
Gender		Gender	
Woman	3 (100%)	Woman	5 (71%)
Man	0 (0%)	Man	2 (29%)
Nonbinary	0 (0%)	Nonbinary	0 (0%)
Prefer not to say	0 (0%)	Prefer not to say	0 (0%)
Other	0 (0%)	Other	0 (0%)
Are you Indigenous?		Are you Indigenous?	
Yes	0 (0%)	Yes	1 (14%)
No	3 (100%)	No	6 (86%)
Are you a visible minority?		Are you a visible minority?	
Yes	0 (0%)	Yes	1 (14%)
No	3 (100%)	No	6 (86%)
		Relationship to patient	
		Patient's spouse or partner	3 (43%)
		Patient's child	3 (43%)
		Patient's parent	0 (0%)
		Patient's sibling	1 (14%)
		Other	0 (0%)

Codesign workshops	
Healthcare providers (*n* = 5)	
	Number (%)
Role	
PC physician	2 (40%)
Nurse (PC)	2 (40%)
Nurse practitioner (PC)	1 (20%)
Social Worker	0 (0%)
Other	0 (0%)
Hospital or community?	
Hospital	0 (0%)
Community	5 (100%)
Both	0 (0%)
Preferred language of communication	
English	5 (100%)
French	0 (0%)
Location	
Urban (more than 1000 people)	4 (80%)
Rural (less than 1000 people)	1 (20%)
Years in practice	
0–9	0 (0%)
10–19	3 (60%)
20–29	1 (20%)
30–39	1 (20%)

Low‐fidelity prototype workshops	
Healthcare providers (*n* = 20)	
	Number (%)
Role	
PC physician	9 (45%)
Nurse	0 (0%)
Nurse practitioner (PC)	1 (5%)
Nurse specialist (PC)	2 (10%)
Social worker	2 (10%)
Home care coordinator	2 (10%)
Home care manager/director	4 (20%)
Hospital or community?	
Hospital	8 (40%)
Community	7 (35%)
Both	5 (25%)

Abbreviations: PC, palliative care.

Through working meetings, the research team identified key findings from the patient/caregiver and HCP codesign workshops including areas of need and areas of intervention. Areas of need were key issues and challenges identified and described by patients and caregivers. The areas of need were classified into four categories (emergency and health needs readiness, logistics of care set‐up, psychosocial preparation and life at home) with subcategories (see summary in Table 2).

**Table 2:** Areas of need from codesign workshops.

Category	Subcategory	Key issues/challenges	Design considerations
Emergency and health needs readiness	Emergency readiness	− Not knowing who to call in an emergency or when to call 911 (phone number for emergency services in Canada/North America). − Not having equipment in place when arriving home becomes an emergency. − Not knowing what to have set up for the transfer home from the hospital.	− Having phone numbers to contact in an emergency, understanding who to call and when to call them. − List of necessary equipment needed in the home before discharge. − Preparation for the transfer from hospital—equipment needed, cost of transportation.
	Health needs readiness	− Needing a kit with medications/supplies to deal with emergencies without having to call 911. − Concerns about DNR and communicating to emergency services.	− Having a symptom management or symptom response kit and/or emergency preparedness kits. − Having confidence that all medical professionals will respect patients' wishes with DNRs.
	Health needs readiness over time	− Concerns about planning for health needs in the future, as the disease progresses.	− Knowing where to get more support as disease progresses.
Logistics of care set up	Home equipment	− Selecting the right equipment for home. − Finding suppliers for needed equipment. − Struggle in connecting with the resources needed with forwarding services. − Not getting home resources in time.	− Having what's needed that fits their home set‐up. − Having a list of local providers for equipment. − What information is needed on supplier? Where to source, and so forth. − Home was set up before discharge.
	Getting home and setting up care at home	− Don't have a home layout that works. − Don't know how to use what at home. − Equipment needs are a surprise and become an emergency.	− Adjusting the home layout so that it meets their needs. − Education sessions to know how to do what. − Know and prepare for what's needed.
	Medical care and protocol	− How to administer all the needed medications. − How to manage the medications. − Is this medication plan going to meet my goals for coming home?	− Familiar with and capable of administering their medications. − Know all the operational issues of their medications. − Discussion, input and plan for medication that will address their goals.
Psychosocial preparation	Preparing for the unknown	− What to expect.	− Preparing for the trajectory.
	Learning caregiving basics	− How to care for someone.	− Basics of caring at home
	Managing change	− How to manage change.	− Make a game plan for care (if this happens, then what)
	How to cope	− Coping when resources fall short. − Lack of communicated supports for caregivers. − Caregiver burden.	− Interventions that help boost patient and caregiver morale. − Knowledge of and access to respite care. − Caregiver feels the ability to keep going (sustainable daily life) and has a variety of supports to help.
Life at home	Being prepared for emergency situations	− Feeling unprepared for future emergency situations.	− Easy access to emergency supplies and contact information, and knowing who to contact in specific emergencies (e.g., an emergency related to a health condition such as a seizure).
	Feeling educated on patient‐care procedures	− Feeling uneducated about patient care requirements once the patient is discharged home.	− Adequate education and hands‐on demonstrations of patient‐care tasks are needed to succeed.
	Navigating the impacts of COVID‐19	− The COVID‐19 pandemic impacts staff availability. − The COVID‐19 pandemic impacts opportunities for social interaction. − The COVID‐19 pandemic impacts the availability of equipment supplies.	− Increased staff availability. − More opportunities to socialize. − Increased availability of equipment.
	Having functional home environments	− The home environment was not suited for the patients' needs once the patient was discharged home.	− Home environments should be evaluated and arranged by OTs for safety and mobility concerns. − Supplies should be easily accessible for both patients and caregivers before patient discharge.
	Communicating the patient's wishes and needs	− In some cases, patients' wishes have not been respected.	− Patients' needs and wishes should be outlined, respected and communicated to all personnel.

Abbreviations: COVID‐19, coronavirus disease 2019; DNR, do not resuscitate; OT, occupational therapist.

The research team then organized the key issues and challenges identified in the codesign workshops into common themes that could be addressed by codesigned interventions, identifying potential design directions. These were classified into three categories (codesigning a transition planning process, codesigning a capacity building process and codesigning a point of contact role) with subcategories (see summary in Table 3).

**Table 3:** Areas of intervention from codesign workshops.

Category	Subcategory	Key issues/challenges	Design considerations
Codesigning a transition planning process	Elements of the plan	− Not knowing what medication is needed and where to fill. − Lack of information on what equipment is needed or where to get equipment. − How to set up the physical home to suit functional needs as well as higher goals of living. − What is needed to get home (e.g., equipment, transportation). − Uncertainty about who to call in an emergency. − Having a network for support.	− Clear communication about medication information and plan for how to get medication. − List of equipment needed and contact information for providers of equipment. − Help from OT to ensure house is set up for arrival. − Preparation for the transfer from hospital, list of equipment needed, information on cost of transportation. − List of emergency contacts and an org chart to understand who does what role, single point of contact when unsure who to contact. − Planning a list of personal contacts that can help with groceries, meals, other errands.
	Communication of the plan	− Telling them when to expect what? − Who spears the discharge plan? How is care plan documented? − Who is the discharge plan shared with?	− Clear communication about what to expect immediately after arriving home, but also as time progresses. Information on who to contact for questions. − Point of contact if anything about the plan is unclear. − Ensuring family/caregivers are present when discharge plan is discussed and opportunity to ask questions.
	Changes to the plan over time	− How will the initial plan change over time?	− Before leaving the hospital, there is a plan in place for who to contact as needs change.
Co‐designing a capacity building process	Lack of information regarding equipment	− Uncertainty around the volume of supplies needed and where to source. − Uncertainty regarding how to install equipment.	− Guidance document on equipment and list of suppliers. − Guidance document on equipment installation.
	Confusion over how to effectively set up the home	− Lack of guidance on how to set up the home.	− Further and earlier involvement of OT.
	Caregiver support needed	− Caregiver burden. − Psychosocial needs of caregiver not met.	− More respite care provided by PSWs. − Social workers or other mental health practitioner to support caregiver.
	Preparing for transition home	− Limited guidance on what to expect and how to care for someone at home.	− Guidance document on home care, training module for caregivers.
	Balancing universality with customizability	− Desire for individualized care program. − Need for an intervention that can be scaled.	− Training tools and personnel intervention with room to customize.
	Communications	− Uncertainty regarding point of contact. − Uncertainty regarding what resources are available. − Confusion reroles of multiple providers.	− Guidance document and delegation of a single point of contact. − Whiteboard on the wall where you can write day of the week/who's calling at what time/who is coming at what time.
Codesigning a point of contact person	Communication challenges	− Medical protocols are unclear. − Unsure which supports are available. − Supplies and equipment information is unclear. − Who will assist with what tasks? − Who assesses the home situation as the condition progresses? − The amount of information received is overwhelming. − Information received was not always consistent.	− Communicate clear medical protocols. − Provide a list of available patient and caregiver supports and services, both in‐person and virtual. − Create a list of required supplies and equipment: Outline where this can be purchased, rented and costs associated with each avenue. − Outline the types of support required, who is designated to each role, their contact information, and how often that person will be in contact with the patient/caregiver (either in‐person or virtual). − Connect with an OT who can complete regular home assessments. − The point of contact person can: 1. Simplify the information content. 2. Reduce the number of streams information is given to patient/caregiver by liaising with healthcare team. − Ensure information provided to the caregiver/patient is accurate.
	Developing a comprehensive care plan	− Care plan should be developed with the patient, and should address the goals of the patient.	− The care plan is developed with the patient, and is tailored to the patient's needs.

Abbreviations: OT, occupational therapist; PSW, personal support worker.

The research team reviewed data from patient and caregiver workshops and established design principles to guide the development of low‐fidelity prototypes. Five design principles were identified: (1) Equip patients and caregivers to live at home in a new way; (2) help patients and caregivers successfully navigate a new system of care at home; (3) utilize an intervention that involves having elements documented in writing (as opposed to a digital solution); (4) build capacity for patients and caregivers to manage the patient's deteriorating condition over time; and (5) equip patients and caregivers to succeed when supports are not sufficient.

Through working meetings, the research team identified areas of focus and considerations (see Table 4) for the concept sketches (Supporting Information S1: Appendix A). The design researcher and communications designer refined the ideas by combining elements into four low‐fidelity prototypes (Figure 5) which included a checklist, quick reference sheets, a patient/caregiver workbook and a transition navigator role.

**Table 4:** Areas of focus for the low‐fidelity prototype concept sketches.

Concept	Description	Design considerations
Back‐to‐home supply list	Outline and contents for a supply list and how‐to list for patients.	− List of equipment and contact information for suppliers. − Supply list and instructions for home supplies. − Outline where equipment can be purchased, rented and costs associated with each avenue. − Future equipment required as patient's needs change. − Conversation guide and sign‐off on completed conversations. − Would involve multiple healthcare providers to complete.
The caregiver checklist	What could a patient/caregiver checklist look like before leaving the hospital?	− Equipment is set up for use during transportation home (e.g., oxygen). − Equipment is set up at home for arrival (e.g., bed, wheelchair, commode, etc.). − List of transportation services. − Would involve multiple healthcare providers to complete even if it is geared towards caregiver.
Medication quick reference sheet	What would be on a worksheet for patients and caregivers to fill out themselves (or with healthcare provider) to be prepared for medicating at home?	− Information on the pharmacy or options for delivery? − Who will get medication if the patient cannot do it themselves? − Clear and accessible way of knowing how to administer medication, hard copy of medication protocols. − Need to work with healthcare team to prepare emergency medications/supplies in a kit for their specific health concerns. − Existing system in hospital, but not always compliant on filling it in.
Patient‐centered organization chart	A comprehensive contact sheet with the information patients and caregivers need.	− Tree diagram of support: contact name/details, picture if possible (patient/caregivers can know what the person looks like), position, tasks they will assist with. − Helpful before discharge to understand who is who and how to get help once arriving home. − Few separate considerations for lists of contacts: (1) general point of contact for when people are unsure who to contact, (2) contacts for equipment set up, (3) emergency contacts, (4) personal contacts for help with errands. − Could be online to have contacts updated regularly.
The tear‐away, carbon‐copied, in‐my‐wallet, on‐my‐fridge call‐card	How could we provide reliable phone numbers in a written way?	− Need list of phone numbers for contacts that can be shared or placed in different locations in the home. − People can share it with all the family/friends. − Who to call after hours. − Hospital can also have the wrong number, how to keep numbers up‐to‐date.
First things first—Immediate training as part of discharge	What would an immediate training session look like when someone transitions to their home?	− Hands‐on caregiver training before patient discharge. − If in‐person training is not available, virtual training sessions could serve as an option (webinars, recordings, playlists, etc.). − Training modules and guidance documents.
Video training modules	What would be the parts of a video tutorial for transitioning? What's would be covered and how?	− Video demonstrations associated with specific tasks (e.g., moving a patient in/out of bed, feeding techniques). − Basic needs training and information. − Informed by lived experience. − Support for caregivers and family.
Paper training modules	What would be the parts of a paper tutorial for transitioning? What's would be covered and how?	− Print out tutorials associated with specific tasks. − Illustrations and photos to explain steps. − Binder or workbook with tabs.
How‐to FAQ	A how‐to guide. What to do when that is not the case?	− How to set up equipment at home for arrival (e.g., bed, wheelchair, commode, etc.). − Who to reach out to for help with medications, emotional support, equipment/supplies, nutritional needs, emergencies and so forth. − Informed by lived experience.
Transition doula	What would this person be doing, and how would they interface with healthcare providers and patients/caregivers (1) in the hospital, (2) while transitioning, and (3) in the home?	− Point of contact is responsible for ensuring information provided is up‐to‐date and accurate. − Single point of contact. − Support for caregiver(s). − Would check‐in at the hospital, at discharge, upon arrival home and after a few days at home.
Patients and caregivers make a predischarge emergency kit	What could be an ‘in case of emergency’ kit for patients/caregivers?	− Create an emergency navigating guide. − Contact numbers/addresses. − Step‐by‐step instructions for general emergency situations as well as personal conditions (e.g., if the patient is prone to seizures). − Instructions on who to call at what times (e.g., friend, paramedic, 911). − List of relevant supplies/location of supplies.
Palliative Performance Scale journey worksheet	What could a worksheet look like that helps patients/caregivers look ahead to how they will experience home as patient functional status diminishes.	− What to expect from the palliative care experience at home. − List of general resources for home care.
Guide to home care	A *day‐in‐the‐life* guide to home care.	− Provide direction on practical questions of day‐to‐day care/caregiving.
How will they know my needs?	How and where can we make it clear to emergency responders and patient/caregiver that patient/caregiver decisions will be respected?	− Way to make it clear to emergency services if a patient has a DNR form. − Document that outlines patient's wishes regarding DNR protocol, end‐of‐life care and preferred location of death. − Copies should be available to provide to paramedics and hospital staff.

Abbreviation: DNR, do not resuscitate.

### 3.3 Stage 3: Low‐fidelity prototype testing

Next, the research team held four low‐fidelity prototype feedback sessions with a total of 20 HCPs. Eight participants worked in hospitals (40%), seven worked in the community (35%) and five worked across settings (25%). Most participants were PC physicians (*n* = 9; 45%), or worked in various home care roles (*n* = 6; 30%). Table 1 provides a summary of the participants in the low‐fidelity prototype sessions.

Participants provided feedback on each prototype (see Supporting Information S1: Appendix B for a summary). Overall, HCPs cautioned about information overload for patients and caregivers at a time where they are already receiving high volumes of information from their healthcare team. For example, a binder is supplied by home care providers on their initial visit to patients' homes and includes information for patients and caregivers. Participants suggested combining the low‐fidelity prototype checklist, quick reference sheets or workbook into a single document.

Many participants raised questions about who would be assisting patients and caregivers in completing the proposed documents. As many HCPs are involved in patient care at different points during the transition process, there may need to be different HCPs involved to complete the checklist, quick reference sheets, or workbook. Participants also suggested the need to clarify whether patients and caregivers would be responsible to complete certain sections, and where there would be space to add notes.

Finally, the transition navigator role was viewed less favourably in comparison to other prototypes. HCPs cautioned that the role contained overlapping scopes of practice with current care coordinators in the hospital and community. Additionally, HCPs were concerned about the long‐term sustainability of the role when it is no longer financially supported by research project.

**Figure 6:**
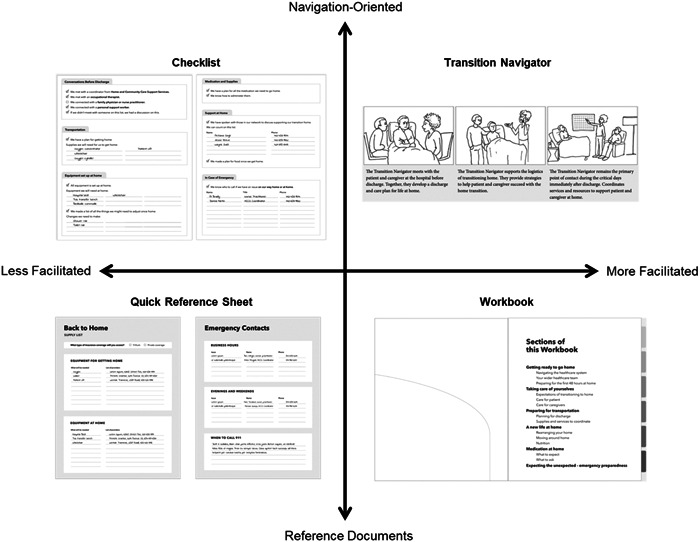
The four prototypes presented in low‐fidelity prototype workshops (checklists, quick reference sheets, workbook and transition navigators) organized into a 2 × 2 grid.

Based on this feedback, the research team sorted the four prototypes along two sets of criteria, with the aim of visually understanding the level of facilitation that would be required for each. A 2 × 2 grid was developed with the vertical axis containing transition navigation‐oriented versus reference prototypes and the horizontal axis depicting prototypes ranging in facilitation requirements from healthcare teams (see Figure 6). The goal was to visually understand the level of facilitation that would be required for each prototype. Therefore, transition navigators were categorized as *navigation‐oriented* and requiring *more facilitation*; checklists as *navigation‐oriented* and requiring *less facilitation*; patient/caregiver workbooks as *reference documents* requiring *more facilitation*; and quick reference sheets as *reference documents* requiring *less facilitation*. This exercise revealed the level of training that would be required for HCPs to be involved in testing and implementing the intervention, especially when considering navigation‐oriented prototypes.

## 4 DISCUSSION

By outlining the codesign and low‐fidelity prototype process at this stage, we hope our findings can support researchers in early stages of codesign and prototype development.

Feedback from patients, caregivers and HCPs in codesign and low‐fidelity prototyping workshops was that simple, targeted documents were best. The research team decided against advancing the transition navigator role for further testing as a high‐fidelity prototype due to concerns from HCPs about its sustainability and challenges in overlap in scopes of practice with existing providers involved in the transition. Despite less favourable feedback HCP participants, this role may be beneficial when long‐term funding is available for the role.

In line with previous research, our study demonstrated the value of codesign in improving healthcare processes. This study combined the perspectives of end users (i.e., patients and caregivers) in the hospital‐to‐home transition and perspectives of stakeholders who have the capacity to create change within the healthcare system. This approach enables alignment of the interventions with patients' and caregivers' needs to improve the experience of transitioning from hospital to home, generating beneficial solutions while working within the constraints of the healthcare system,^21^, ^44^ particularly with buy‐in from HCPs and administrators.^22^, ^27^

The amount of input that was sought from HCPs was higher than in most patient‐centered processes. It was, however, important to engage HCPs at various points in the codesign process to tailor the interventions to a multifaceted healthcare system and ensure interventions' sustainability.^22^, ^27^, ^45^ This codesign process required a balance between occasionally competing viewpoints of the stakeholder groups involved. Taking a patient‐ and caregiver‐centered design approach, the needs of all stakeholders can be met while mitigating competing perspectives.^46^ For example, there was incongruence between patient/caregiver and HCPs' feedback on using paper handouts for checklists, quick reference sheets or workbooks. During low‐fidelity prototyping sessions, HCPs raised concerns about information overload; however, patients and caregivers in codesign workshops voiced that existing resources did not adequately meet their needs and desired more information documented in writing with physical copies provided. As previously highlighted,^44^ HCP perspectives can often take precedence over those of patients and caregivers.^26^, ^47^ We mitigated potential bias by ensuring that the voices of patients and caregivers were always at the forefront of discussions about intervention prototypes.

Many common elements of codesign were adjusted for this project. While in many contexts, codesign involves designing with the community of ‘end‐users’,^48^, ^49^ due to the progressive and debilitating nature of life‐limiting illnesses, codesign in this project involved patients who would never experience the final design. Therefore, codesign outputs were required to stand alone rather than requiring continuation of the design process over time.^50^ Long‐term inclusion of patient and caregiver advisors in this project will ensure future work is patient‐ and caregiver‐centred.^23^

Additionally, codesign often involves group, in‐person sessions and tactile materials, leading to an energetic and creatively rich interaction amongst peers.^48^, ^49^ Rather than holding codesign sessions with many participants, workshops were held with a single patient, caregiver or a dyad on their own. This intimate context permitted focus on individuals' unique experiences. Codesign workshops typically comprise several hours, including open time for free play and experimentation. Given the finite time and energy level available to participants with life‐limiting diseases,^51^, ^52^ sessions were limited to 60 min, workshops were conducted earlier in the day when participants had more energy to participate, and online sessions aimed to remove the burden of transport to and from a codesign session for functionally limited participants. Codesign took the form of speculation, conversation and feedback to proposed ideas and interventions,^48^, ^49^ with participants describing and remodeling proposals to meet their needs without the need to utilize more energetically‐taxing means of artistic expression; this allowed for participants with various levels of motor control and mobility to fully participate in the codesign process.^39^, ^40^, ^48^, ^49^

### 4.1 Strengths

This project's most notable strength was the involvement of patients, caregivers and HCPs at specific points in the codesign process. Involvement of patients and caregivers in the codesign workshops highlighted the components needed to improve care transitions from their perspective. Additionally, feedback from HCPs and administrators in low‐fidelity prototype workshops ascertained how potential interventions would realistically work with clinical workflow, and how to begin tailoring interventions to regional contexts. HCPs and administrators were consulted to ensure the intervention will be tailored to a multifaceted healthcare system, while ensuring patients and caregivers are at the centre of this process. This was demonstrated through the facilitation of online workshops ensuring functionally limited participants or participants with lower energy levels could participate.

Another strength of this study was the application of the Double Diamond Framework^32^, ^33^ to frame the phases of our process,^32^, ^33^ thereby demonstrating how to customize the well‐established framework to fit the goals of a codesign project.

### 4.2 Limitations

Two challenges experienced by the research team included recruitment of patients receiving a palliative approach to care and their caregivers and codesigning an intervention during COVID‐19.

Due to low recruitment numbers, all eligible potential participants were accepted, rather than screening for a more diverse set of participants. This resulted in participants who were primarily White, English‐speaking and of a higher socioeconomic status. Furthermore, the study did not note participants' education levels and literacy skills, which may impact one's preferences for receiving information. From an equity‐informed perspective, some patients and caregivers from marginalized groups may face additional barriers to participating due to low‐income, limited English proficiency, or negative experiences with the healthcare system.^23^, ^53^, ^54^ Furthermore, the perspectives of the participants included in this study may not be fully representative of patient and caregiver needs, particularly of other ethnocultural and/or religious groups, and should not be generalized to all patients and caregivers.

Workshops were initially intended to be conducted in‐person; however, during recruitment, in‐person research was restricted due to COVID‐19 and many participants preferred participating virtually. The pandemic's shortage of community healthcare workers to support patients and caregivers at home was also prevalent across patient, caregiver and HCP's experiences. Therefore, the COVID‐19 pandemic may have impacted all stakeholders' experiences with hospital‐to‐home transitions.

### 4.3 Next steps

We will complete codesign workshops and low‐fidelity prototype testing at a second site, Bruyère Continuing Care in Ottawa, Ontario, Canada. The low‐fidelity prototypes from both sites will be refined using the feedback from workshop participants and will be developed into high‐fidelity prototypes. High‐fidelity prototypes will be presented to patients, caregivers and HCPs at both sites to further improve intervention feasibility. The high‐fidelity ACEPATH prototype will undergo an accessibility assessment using accessible design principles.^39^, ^40^

An implementation study will then be conducted to evaluate acceptability, appropriateness, feasibility, costs and fidelity of ACEPATH. Next steps will thus address the Deliver—Implementation Phase through high‐fidelity prototype testing and implementation testing.

## 5 CONCLUSION

This paper provides a detailed process for codesigning key components of an intervention aimed at improving the hospital‐to‐home transition for patients receiving a palliative approach to care and their caregivers. The finalized ACEPATH intervention and the steps taken to refine it will be covered in a subsequent manuscript. This methodology demonstrates means of engaging patients and caregivers throughout the development of interventions, and HCPs in the intervention's fit within an evolving healthcare system.

## AUTHOR CONTRIBUTIONS

**Madeline McCoy**: Formal analysis; investigation; project administration; writing ‐ review & editing; visualization; writing—original draft. **Taylor Shorting**: Formal analysis; investigation; project administration; visualization; writing—review and editing. **Vinay Kumar Mysore**: Formal analysis; investigation; methodology; project administration; visualization; writing—review and editing. **Edward Fitzgibbon**: Conceptualization; formal analysis; funding acquisition; investigation; project administration; visualization; writing—review and editing. **Jill Rice**: Conceptualization; formal analysis; funding acquisition; investigation; project administration; visualization; writing—review and editing. **Meghan Savigny**: Formal analysis; investigation; visualization; writing—review and editing. **Marianne Weiss**: Formal analysis; investigation; visualization; writing—review and editing; methodology. **Daniel Vincent**: Formal analysis; investigation; visualization; writing—review and editing. **Meaghen Hagarty**: Formal analysis; investigation; visualization; writing—review and editing. **Krystal K. MacLeod**: Formal analysis; investigation; visualization; writing—review and editing. **Natalie C. Ernecoff**: Formal analysis; investigation; methodology; visualization; writing—review and editing. **Rex Pattison**: Visualization; writing—review and editing. **Mona Kornberg**: Visualization; writing—review and editing. **Adrianna Bruni**: Visualization; writing—review and editing. **Shirley H. Bush**: Formal analysis; visualization; writing—review and editing. **Kerry Kuluski**: Visualization; writing—review and editing. **Valerie Fiset**: Visualization; writing—review and editing. **Cecilia Li**: Visualization; writing—review and editing. **Henrique A. Parsons**: Visualization; writing—review and editing. **Geneviève Lalumière**: Investigation; formal analysis; visualization; writing—review and editing. **Tara Connolly**: Visualization; writing—review and editing. **Colleen Webber**: Visualization; writing—review and editing. **Sarina R. Isenberg**: Conceptualization; formal analysis; funding acquisition; investigation; methodology; project administration; supervision; visualization; writing—review and editing.

## CONFLICT OF INTEREST STATEMENT

The authors declare no conflicts of interest.

## ETHICS STATEMENT

The Ottawa Health Science Network Research Ethics Board (OHSN‐REB) and the Bruyère Research Ethics Board (BREB) determined that this project falls within the context of quality initiative, quality improvement, quality assurance and/or program evaluation and have exempted this project from requiring ethics approval.

## Supporting information

Supporting information.

## Data Availability

The data that support the findings of this study are available from the corresponding author upon reasonable request.
