# Supplementary material for: Advancing the Care Experience for patients receiving Palliative care as they Transition from hospital to Home (ACEPATH): Codesigning an intervention to improve patient and family caregiver experiences
Source: Health Expect. 2024 Mar 28;27(2):e14002. doi: 10.1111/hex.14002 (PMC10979115; doi:10.1111/hex.14002)
Supplement: Supplementary file 1 — Supporting information. [file HEX-27-e14002-s001.docx]

# Appendices

## Appendix A

Sketches of prototypes and storyboards before synthesizing into low-fidelity prototypes.


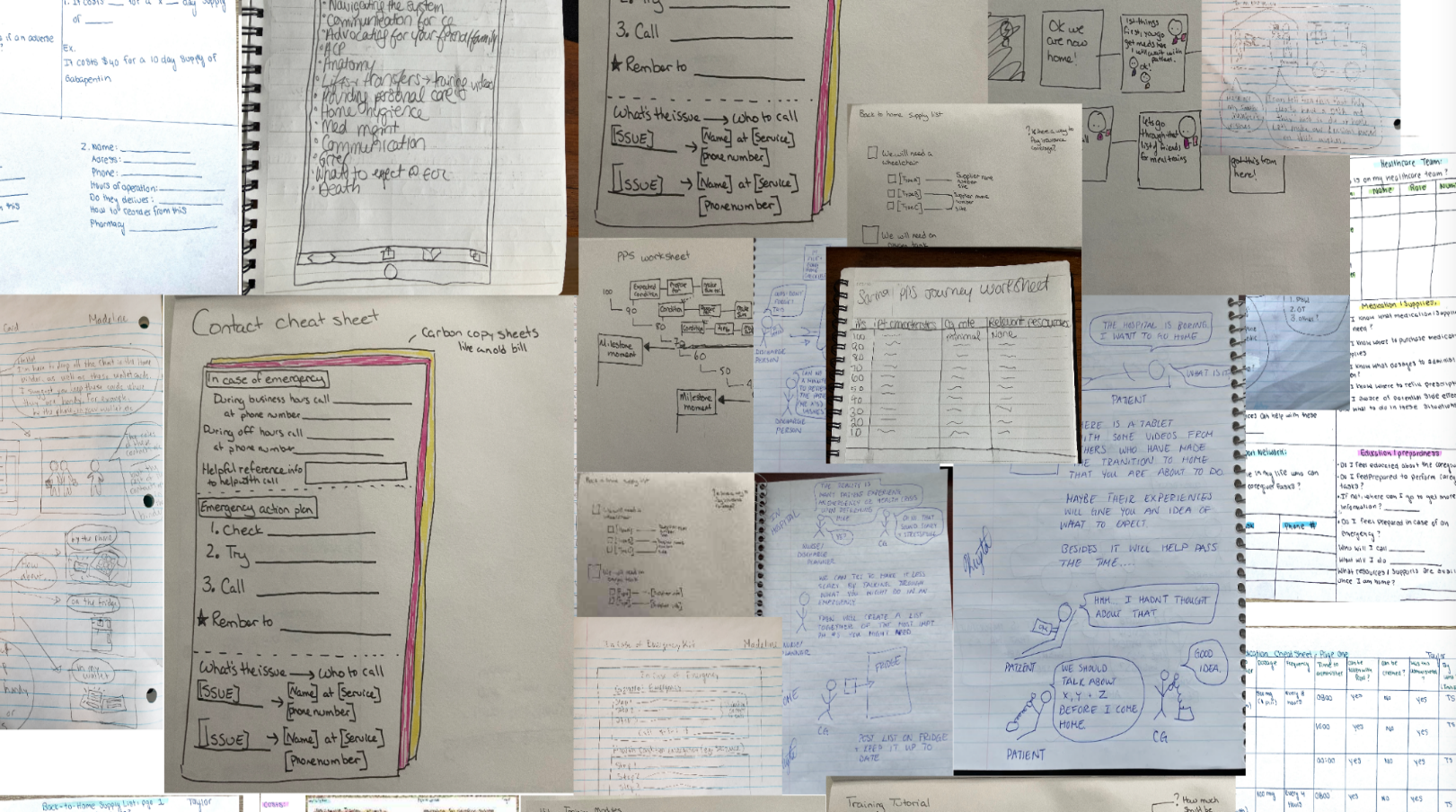


## Appendix B

Detailed breakdown of feedback from low-fidelity prototype sessions

### Checklist

*What does the checklist look like?*

- How can this checklist be customizable to the patient? Can some items be completed in-hospital or at home?
- How does the checklist identify which services are covered by private insurance and/or are government funded?
- What happens if the checklist is not fully completed prior to discharge? Some patients might not need to see specific providers before discharge or at all, who explains to patients/families that a step might not the be completed in their case and why?
- Suggestions to add to the checklist:
  - Do you have a symptom response kit?
  - Do you have backup medications for an emergency?
  - Do you know when medications will be received?
  - Do you know which pharmacy will be filling the mediation?
  - Who is the primary contact for the patient?
  - Do patients/caregivers feel confident and prepared to be discharged home?

*What problems is the checklist is trying to solve?*

- Identify which healthcare providers have had conversations with the patient/family
- The checklist can be used to remind healthcare providers of the steps that should be taken prior to discharging a patient. Currently, there are some conversations that do not happen prior to discharge.
- Ensures that medications have been reviewed with patient and caregiver before discharge.

*What are the concerns about the checklist?*

- Checklists do not emphasize how long it may take for services to be set up in the home
- How can this checklist be adapted for those with accessibility barriers (e.g., translated into other languages, unable to read, different levels of vision)?
- How does this checklist get completed when patient discharges occur on the weekend?
- Once discharged home, the information on the checklist is probably out of date
- How do you ensure that there are not too many handouts for patients and caregivers?

### Quick Reference Sheet

*What do the quick reference sheets look like?*

- How can quick reference sheets be individualized?
- What happens when information changes and/or needs to be updated?
- Need to ensure that multiple copies are accessible to patients/families

*What problems are the quick reference sheets trying to solve?*

- To bridge the gap between being discharged and connecting with a care coordinator in the community
- To supplement information in Chart in the Home binder
- To empower patients/families

*What are the concerns about the quick reference sheets?*

- Too much information can be overwhelming

### Patient/Caregiver Workbook

*What does this workbook look like?*

- How can the workbook be tailored to each patient being discharged?
- Is this a resource that everyone receives?
- Will this workbook repeat information that is already provided by other resources? Could an existing resource be modified to be used in this context?

*What problems is this workbook is trying to solve?*

- To supplement what resources already exist
- To empower patients and caregivers

*What are the concerns about this workbook?*

- It is difficult to reference such a detailed booklet in an emergency situation
- Families may prefer to use their own workbook

### Transition Navigator

*What does this role look like?*

- What are the time requirements to properly execute this role in relation to case load?
- Can this role be virtual?
- Who fulfills this role, e.g., social worker, Home and Community Care Support Services (HCCSS) coordinator, Registered Practical Nurse (RPN), Registered Nurse (RN)?
  - This role contains both psychosocial and medical responsibilities – who is qualified to complete this role?
  - The person fulfilling this role must be familiar with surrounding systems in both the hospital and community. Could the person fulfilling the role have a hospital connection and then familiarity with the community?
- Does this person work in both community and hospital settings?
  - If this role spans across both settings, where is the boss/paymaster housed?
- What is the timeframe of patient/family engagement?
  - When are individuals introduced within the hospital?
  - When does follow-up end within the community?
- How does this role operate when patients require care outside of standard hours (e.g., after-hours care, emergency room discharges, weekend transitions)?

*What problems is this role is trying to solve?*

- The need for seamless communication between healthcare providers in different care settings across hospital and community
  - Bridge the gap between HCCSS hospital and community care coordinators
  - Bridge the gap between care provided by the social worker in the hospital and community care. Social workers do not have capacity to carry out follow-up care outside of their scope and many patients do not have a social worker in the hospital
- Increased efficiencies within the healthcare system
- Assisting individuals who are healthcare illiterate
- To advocate for those who cannot advocate for themselves and/or do not have support to advocate for themselves

*What are the concerns about this role?*

- It is resource intensive when taking caseload intensity into consideration
- What is the sustainability of this role long term?
- What is the scope of practice of this role, in relation to in-hospital and community HCCSS coordinators?
- Accountability concerns, who does this role report to?
